# Dexterous Identification of Carcinoma through ColoRectalCADx with Dichotomous Fusion CNN and UNet Semantic Segmentation

**DOI:** 10.1155/2022/4325412

**Published:** 2022-10-10

**Authors:** Akella S. Narasimha Raju, Kayalvizhi Jayavel, Thulasi Rajalakshmi

**Affiliations:** ^1^Department of Networking and Communications, School of Computing, SRM Institute of Science and Technology, Kattankulathur, Chennai 603203, India; ^2^Department of Electronics and Communication Engineering, School of Electrical and Electronics Engineering, SRM Institute of Science and Technology, Kattankulathur, Chennai 603203, India

## Abstract

Human colorectal disorders in the digestive tract are recognized by reference colonoscopy. The current system recognizes cancer through a three-stage system that utilizes two sets of colonoscopy data. However, identifying polyps by visualization has not been addressed. The proposed system is a five-stage system called ColoRectalCADx, which provides three publicly accessible datasets as input data for cancer detection. The three main datasets are CVC Clinic DB, Kvasir2, and Hyper Kvasir. After the image preprocessing stages, system experiments were performed with the seven prominent convolutional neural networks (CNNs) (end-to-end) and nine fusion CNN models to extract the spatial features. Afterwards, the end-to-end CNN and fusion features are executed. These features are derived from Discrete Wavelet Transform (DWT) and Vector Support Machine (SVM) classification, that was used to retrieve time and spatial frequency features. Experimentally, the results were obtained for five stages. For each of the three datasets, from stage 1 to stage 3, end-to-end CNN, DenseNet-201 obtained the best testing accuracy (98%, 87%, 84%), ((98%, 97%), (87%, 87%), (84%, 84%)), ((99.03%, 99%), (88.45%, 88%), (83.61%, 84%)). For each of the three datasets, from stage 2, CNN DaRD-22 fusion obtained the optimal test accuracy ((93%, 97%) (82%, 84%), (69%, 57%)). And for stage 4, ADaRDEV^2^-22 fusion achieved the best test accuracy ((95.73%, 94%), (81.20%, 81%), (72.56%, 58%)). For the input image segmentation datasets CVC Clinc-Seg, KvasirSeg, and Hyper Kvasir, malignant polyps were identified with the UNet CNN model. Here, the loss score datasets (CVC clinic DB was 0.7842, Kvasir2 was 0.6977, and Hyper Kvasir was 0.6910) were obtained.

## 1. Introduction

Health is of utmost importance for mankind. Good health practices are essential for the survival of the human race. However, owing to environmental pollution and personal habits of human beings, their health is adversely affected [1]. According to statistics from various Indian health organizations, 22% of patients seek medical treatment at least thrice a year for related health problems. Carcinomas are ranked as the most important health disorder and a hazardous evil [2].

The carcinoma affects the inner cell of the human body and develops exponentially, damaging the entire affected organ. Progressive growth damage to the human body can lead to life-threatening situations. There are many types of carcinomas that affect organs [3]. Cancers that are hazardous to human organs include breast cancer, prostate cancer, cancer of Basel cells, skin cancer (melanoma), lung cancer, leukemia, lymphoma, and colon cancer. This dangerous affliction invades organ tissues and causes potentially fatal conditions [4]. Furthermore, blood cells are ineffective in defending against this malady and cannot protect organs from damage.

Colorectal carcinoma is the most hazardous and irritable disease of the gastrointestinal tract. This condition can have devastating effects on a person's daily routine. This disease affects food digestion and creates severe gastric problems, which then create critical bowel problems. This cancer is caused by human eating and consumption patterns. The disease is associated with the brain as too many psychological thoughts can also affect the human colon. Seven lakh people are diagnosed annually with colorectal cancer, and the morality rate is approximately 525,000 worldwide [5]. Those suffering from severe problems, such as gastric issues, aged 50 years and over must get themselves tested via colonoscopy screening technology.

Colonoscopy is the most advanced screening technology and is 95% acceptable. This test method, with a number of video graphs and frames captured, examines the entire large intestine, which is approximately five feet long and three inches wide. In the videos and images captured using this technique, every inch is scanned for disease diagnosis [6]. This screening procedure is performed by gastroenterologists, and videos are obtained and photographic images are analyzed by radiologists. The average acquisition time is between 30 minutes and one hour. Early detection and rapid diagnosis of these medical conditions provide the appropriate solutions for treatment [7–10].

The videos and the photographic images obtained thus are presented as datasets. Researchers conducting colorectal cancer research organize data and store them on a website for public access. The computer-aided diagnosis system (CADx) [11, 12] diagnoses health problems using computers with technologies such as artificial intelligence (AI). Deep learning (DL) is a subset of AI technology that is key in CADx systems, with which every image is skillfully considered and the image characteristics are extracted for further experimentation. Publicly available colonoscopy datasets such as CVC Clinic DB, Kvasir2, and Hyper Kvasir provide inputs for CADx [13, 14]. CADx works with the appropriate deep learning technologies (DLTs). The system referenced in this article is ColoRectalCADx.

The ColoRectalCADx system works entirely with DL that can recognize carcinoma using an innovative architecture. For medical colonoscopy motion images, we use CNN as a suitable network [15]. CNN works well as a classifier and feature extractor. This article demonstrates how the key elements of the system elaborately interact with CNN [16, 17].

The main objective of this research study is as follows:

- Design the colorectal cancer recognition system through a five-stage system.
- The early stage of detection is the classification of images through a colonoscopy using seven different CNNs.
- The next phase of the system presents nine fusion models of the CNN and obtains the most accurate model for cancer classification using three datasets.
- In addition, end-to-end CNN and fusion models represent transfer learning with discrete transform wavelet (DWT) and support vector machine classification (SVM). This classification determines the most appropriate model for cancer recognition.
- To accurately identify a cancer polyp for malignant recognition, a visualization technique such as semantic segmentation using the UNet CNN model is used in a later stage.

### 1.1. Organization of the Study

The rest of the study is organized as follows.  Section 2 provides an overall literature review and Section 3 provides the materials and explains the methods.  Section 4 discusses the results and Section 5 concludes the study.

## 2. Related Work

Previously, colorectal cancer was identified using different approaches. In these approaches, an architecture had to be developed to build a system. This system considered various elements of the structure. For every structure, the documentation related to the concept must be examined. Different articles are reviewed on the different blocks with their merits and demerits. The optimal approach is coming up with an appropriate article to identify colorectal cancer.  Table 1 represents the literature related to the addressed concept.

## 3. Materials and Methods

The main objective is to build a system to uncover colorectal disease as carcinoma with automatic and skilled recognition of eases.

**Figure 1:**
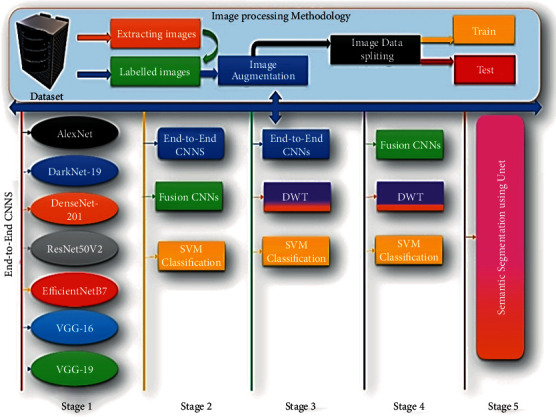
The flow diagram of the colorectalCADx system.

Flow diagram: the flow diagram for the proposed ColoRectalCADx [28, 29] system with the explanation of each stage is shown in Figure 1.

The three remarkable datasets that are backed up to the local servers are recovered, and each dataset is saved in a particular folder as CVC Clinic DB, Kvasir2, and Hyper Kvasir. These files constitute 2, 8, and 23 classes corresponding to their labels. Labeled folders contain colonoscopy images. As the recovered images are in various image sizes, they are resized into the 224 × 224 pixels size images using the inputs for CNN experiments. These augmented techniques are applied to resize images, with a zoom range of 0.4, a rotation range of 15, and a horizontal flip. Augmentation enhances the image quality of the input image. In additionally, the input images in the 2-, 8-, and 23- class datasets are divided into training and testing datasets with a 70 : 30 ratio:

- In addition, the proposed automatic and effective CADx system called ColoRectalCADx is entirely dependent on the five stages for classifying and discovering colorectal carcinoma polyps.
- The first stage classifies and extracts the features of the seven end-to-end CNNs.
- In the second stage, CNNs are responsible from end to end and fusion CNNs are associated with classification using support vector machines (SVM). SVMs are effective for large dimensions and multiclass problems. The kernel function used to map the characteristic space into a new domain that can easily discriminate between classes of a dataset. Therefore, it is widely used with the huge DL feature dimension, extracted from CNN.
- The third stage is transfer learning of various end-to-end CNNs with DWT, which is combined with the SVM multi-class classification for extracting temporal and spatial features.
- The fourth stage is the transfer learning of the fusion CNNs with DWT, followed by combining with the SVM multi-class classification for extracting temporal and spatial features.
- At each stage, performance parameters such as accuracy are recovered and compared with other parameters, and the best model for the ColoRectalCADx system is found.
- The fifth stage is the most efficient technique for semantic segmentation of input images and involves identifying the exact malignant polyp with the UNet CNN model [30].

**Figure 2:**
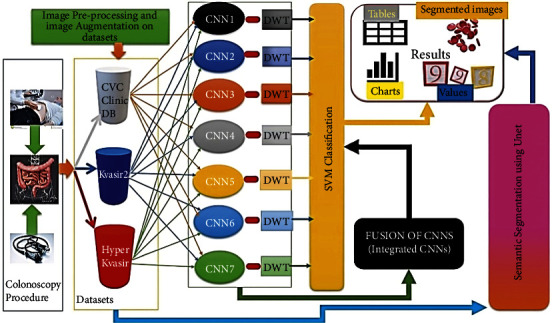
Proposed colorectalCADx block diagram.

The CADx is designated as ColoRectalCADx, which is developed for carcinoma recognition block diagram, as illustrated in Figure 2, and the detailed explanation as follows in a step-by-step manner.

### 3.1. Colonoscopy

People with gastrointestinal problems are referred by a gastroenterologist, who may suggest the colonoscopy procedure to diagnose the disease. Colonoscopy is the procedure of screening the entire large intestine under local anesthesia administered to the patient. The procedure inserts the illuminated colonoscope equipped with the front-end camera into the large intestine through the rectum. It takes videography and photographs at various positions throughout the large colon and a certain area of the small colon [31]. This procedure takes about an hour. The obtained videos and the photographic images are analyzed and saved on local servers.

### 3.2. Dataset

There are input datasets to support deep learning studies. This study uses publicly accessible datasets. The ColoRectalCADx system uses three datasets labeled as CVC-Clinic DB, Kvasir2, and Hyper Kvasir labeled.

**Figure 3:**
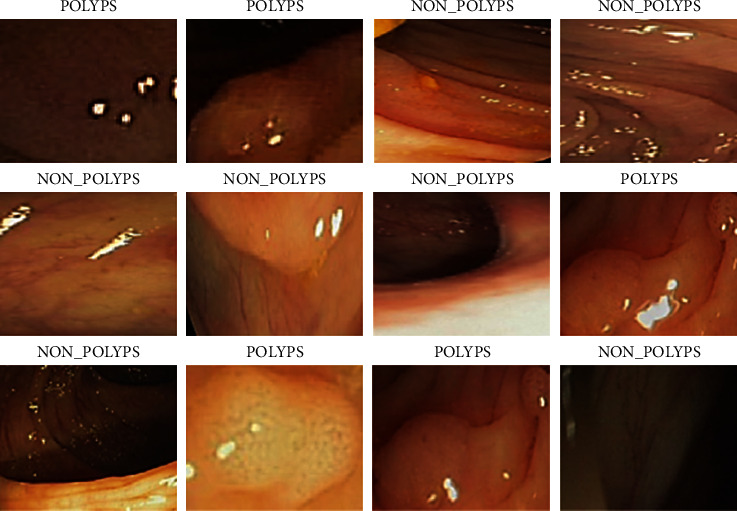
CVC clinic DB sample dataset.

The CVC Clinic DB dataset included 2 classes such as labeled polyps and nonpolyp [32]. The sample images of the CVC Clinic DB dataset are shown in Figure 3.

**Figure 4:**
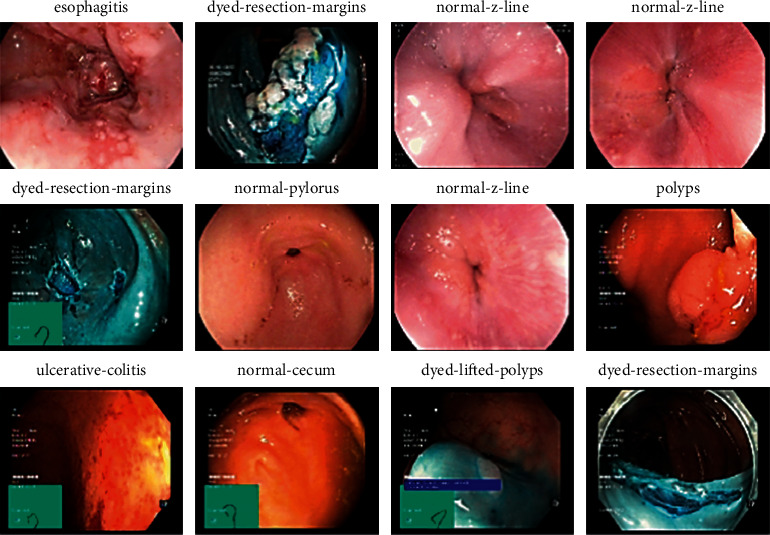
Kvasir2 sample dataset.

The Kvasir2 dataset includes eight labeled classes, labeled as Dyed-Lifted Polyps, Dyed-Resection Margins, Esophagitis, Normal-cecum, Normal-cecum, Normal-z-line, Polyps, and Ulcerative Colitis [33, 34]. The sample images of the Kvasir2 dataset are shown in Figure 4.

**Figure 5:**
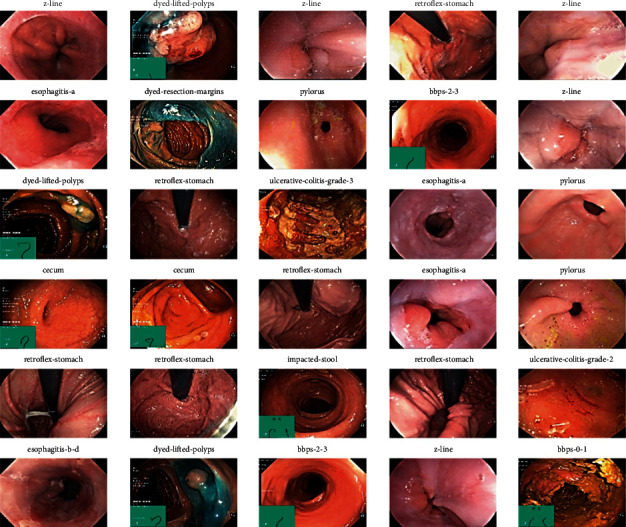
Hyper kvasir sample dataset.

The Hyper Kvasir Labeled dataset includes as lower GI tract and upper GI tract and these two classes of datasets are further classified and labeled as 23 classes named as barrettes, barrettes-short-segment bbps-0-1, bbps-2-3, cecum, dyed-lifted-polyps, esophagitis-a, esophagitis-b-d, hemorrhoids, esophagitis-a, ileum, impacted-stool, polyps, pylorus, retroflex-rectum, retroflex-stomach, ulcerative-colitis-grade-0-1, ulcerative-colitis-grade-1, ulcerative-colitis-grade-1-2, ulcerative-colitis-grade-2, ulcerative-colitis-grade-2-3, ulcerative-colitis-grade-3, z-line [35, 36]. The sample images of the Hyper Kvasir dataset are shown in Figure 5.

Each labeled class has different number of images of different sizes. The various image sizes are scaled to 224 × 224 pixels. This image size is provided as input to the next stage of the CNN experimental procedures. The dataset is divided into training and testing with the 70 : 30 ratio.

### 3.3. Convolutional Neural Networks

**Figure 6:**
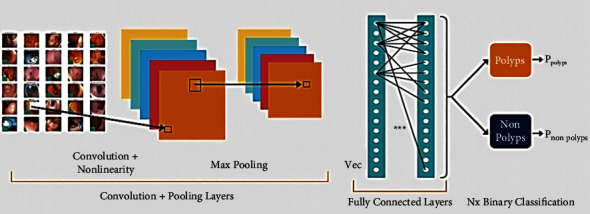
CNN architecture.

CNNs are used to solve classification problems in healthcare computing. They play a key role as the main element of the ColoRectalCADx system. This system works entirely with CNN, whose Figure 6depicts input data images, convolution, pooling, activation, dropout, and fully connected layers [37–40]. This system elaborately works with seven different pretrained CNNs as end-to-end CNNs. They are AlexNet, DarkNet-19, ResNet-50V2, DenseNet-201, EfficientNetB7, VGG-16, and VGG-19. In addition to these end-to-end CNNs, the fusion of these end-to-end CNNs is being considered for further experimentation. Every fusion CNN is worked as the one specified model [41–45]. Here nine fusion models are presented in Table 2 with their suggested new names.

Each CNN fusion model combines end-to-end CNN models [46], and the combination is used for later experiments involving training and testing. Short names are suggested for each fusion pattern for significant comprehension, and these names are used throughout the article. The CNN used for the classification of the input image datasets is illustrated in Figure 6. CNN input is taken as input image datasets; these are CVC Clinic DB, Kvasir2, and Hyper Kvasir images. These images are applied to the convolution layer to retrieve the features of the images. Furthermore, the image features obtained from the previous layer are sent to the maximum pooling layer to filter the image values. Inthe fully connected neural network. Lastly, the final layer is the SoftMax layer to classify the multi-class classification in order to distinguish the classes in the input images. It is possible to find whether the input image is polyp or nonpolyp.

Each end-to-end and fusion model has a specific advantage in the classification of the input medical colonoscopy motion images. This DL technique is advantageous for recognizing colorectal carcinomas. This provides the key perceptional view to identify the diseases effectively and efficiently. A recent study has found that CNNs can be far deeper, more precise, and efficient for learning where smaller connections are made between the layers near the input and those adjacent to the output. The number of total parameters for the end-to-end and proposed fusion CNNs and the number of trainable parameters are presented in Table 3.

The experimental activity with the proposed ColoRectalCADx system involved the system with the oldest and most efficient CNN model AlexNet to the latest EfficientNetB7 model, and experiments that involved fewer layers to the highest number of layers.

All experiments on end-to-end and dichotomous fusion CNNs applied transfer learning for further exploration to extract features. The CNN features map the captured results by applying filters to a dataset input image. In transfer learning, one of the network layers is transferred and replaced with other. Transfer learning implies using the pertinent parts of a predetermined machine learning (ML) model and applying it to a new problem. For the model to work, new aspects are added to solve a specific task. With the transfer of a layer, CNN performance changes in the form of classification results. The main evidence of the transfer of learning is a model formed on one dataset and transferring one's knowledge to another [57–60]. To recognize objects with a CNN, the primary convolutional layers of the network are restricted, forming only the last layers that make a prediction.

### 3.4. Discrete Wavelet Transform (DWT)

The DWT is a discretely transformed wavelet [61, 62]. The wavelet transform breaks down a function into wavelets. A wavelet is a wave oscillation that is localized through time. Its properties include scale and location. The scale sets the wavelet “frequency” and the location sets the wavelet “time.” Frequency is inversely proportional to time. Scale is represented in squished and stretched format. The wavelets distinguish themselves as continuous and discrete. The formula for the discrete wavelet transform is as follows:

(1) $$\begin{array}{c} {T\_\left(m,n\right)=\int }_{-\infty }^{\infty }x\left(t\right)\varphi_{m,n}\left(t\right)dt\text{,} \end{array}$$

where *T*_*m*,*n*_ is the time function of the DWT and *x* (*t*) is the time period.

**Figure 7:**
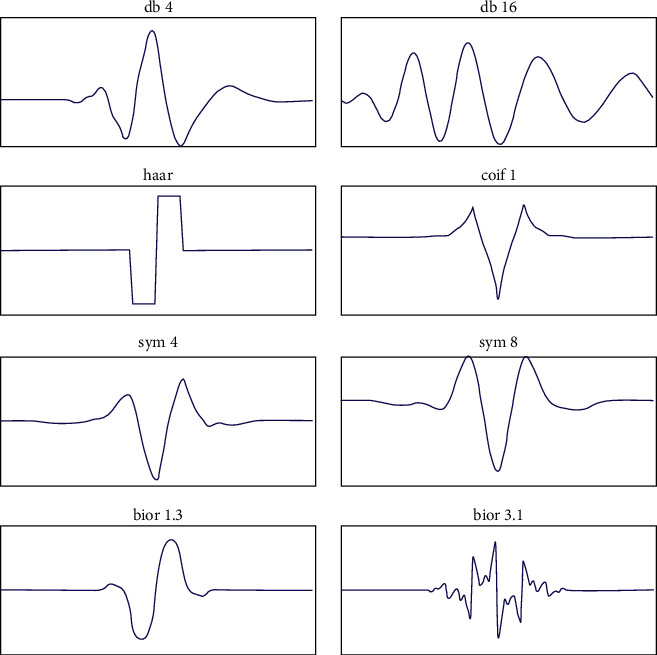
Families belonging to the DWT.

Discrete wavelet transforms can retrieve local spectral and temporal information simultaneously. Functionally, DWTs are represented with different kinds of characteristic forms of access and depend on the application. The characteristic functional forms are depicted and shown in Figure 7. The figure depicts the Discrete Wavelet Transform (DWT) family. The DWT family is classified as Haar, Daubechies, Coeflet, and Discrete Meyer. Haar is the easiest and the squarest waved. Daubéhies wavelets are continuous and asymmetric waveforms. Coeflet is a symmetric waveform. Discrete Meyer wavelets are continuous and symmetric. In all of the abovementioned forms for our experimentation, the square wave “haar” was used to extract the features.

The ColoRectalCADx system works with each CNN from end to end, while fusion is transfer learning with DWT. Minimizing features is an important procedure in input image datasets for medical colonoscopy. It is the essential stage for the transfer learning process to reduce features. DWT is applied in the retrieval of spatial and temporal image features from input images. This application concept removes the Max Pool layer from the CNN and replaces it with DWT. This can concatenate all the different DWT outputs CA (approximation coefficient vector) and CD (detail coefficient vector), and they are depicted as low-pass and high-pass wavelet signals and combined into one channel. Continuous input image signals are considered, and the system transfers the CNN layers into the DWTs and finds the best precision from all CNN models. Then, the DWT “haar” family is considered for the CNN training. Furthermore, the output of the DWT is applied to the SVM for the multi-class classification process.

### 3.5. Support Vector Machines (SVMs)

SVM is an algorithm in ML under supervised learning used for classification, regression, and selection of outliers. This algorithm creates the hyperplane that separates the data into various classes. It selects a hyperplane with the maximum possible boundary between media vectors within the given dataset. The SVM recovers the maximum marginal hyperplane. It further generates hyperplanes for enhanced class isolation. It works on binary classification and multiclass classification [63–65].

In the ColoRectalCADx system, the CNN must convert to SVM. Inside a parameter named kernel_regularizer, the l2 standard is used, and the linear function is passed as the activation function in the final output layer. For multi-class classification, we should use SoftMax as an activation function for SVM [66–68]. The application of the loss is the “squared hinge” for the multiclass classification. Therefore, the last layers of the CNN are responsible for the changes; the linear SVM is represented, and the final accuracies of all the CNN from end-to-end and fusion are obtained.

### 3.6. Semantic Segmentation

In the semantic segmentation of an image, each pixel of an item belongs to the special class to which the same label is assigned. This task categorizes each pixel into an image with preset classes. Semantic segmentation depends on the mask concept, including edge detection. It brings together parts of the image belonging to the same class.

The ColoRectalCADx system integrates the UNet architecture with data scaling and patch extraction with the three Clinic-Seg, KvasirSeg, and Hyper Kvasir colonoscopy datasets to extract malignant polyps. This system can achieve an overall high accuracy for polyp detection, suggesting the importance of using UNet CNN structure with the necessary hyperparameters.

**Figure 8:**
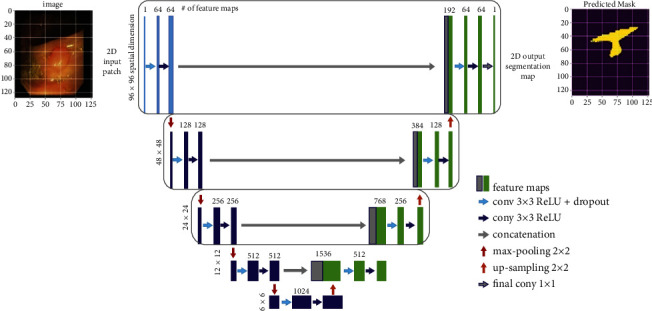
Semantic segmentation using UNet.

In the proposed ColoRectalCADx system, U-net is used to segment medical colonoscopy motion images [69]. The UNet structure for semantic segmentation is shown in Figure 8. Its structure may be widely assumed to be a tail encoder network by a decoder network. Semantic segmentation is the outcome of this network:

- The encoder is the beginning of the framework. Typically, it is a pretrained classification network [70]; it applies convolution blocks trailed by a pooling, which is max pooling, and down samples to encode the input colonoscopy medical motion images into feature depictions at multiple different levels.
- The decoder is the latter end of the frame. It semantically projects the discriminatory characteristics (lower resolution) learned by the encoder on the pixel space, resulting in higher image pixels to obtain a solid classification. The decoder involves up sampling and concatenating followed by coherent convolution processes.

Up sampling in CNN is used for classification and object detection architecture, to reinstate the reduced feature map to the actual original size of the medical colonoscopy motion images, and consequently increase the feature dimensions. Up sampling is also discussed for transposed convolution, up convolution, or deconvolution.

The results of the investigation using the proposed ColoRectalCADx system thus far are presented herein. All experiments are conducted with the system hardware specifications and the software used for the work is presented in Table 4.

In the proposed ColoRectalCaDx system, datasets are an essential component. The datasets used are CVC Clinic DB, Kvasir, and Hyper Kvasir, and the datasets are depicted with 2, 8, and 23 classes, respectively. Each class stores medical colonoscopy motion images, which are accessible for CNN training. Details of the datasets and hyperparameters are presented in Table 5.

For all datasets, experimental research with end-to-end and fusion CNNs are also tested with transfer learning by DWT, followed by SVMs. For experiments with adjusted hyperparameters, the specific hyperparameters for the entire ColoRectalCADx system are provided in Table 6.

## 4. Results

The ColoRectalCADx system comprises five stages. In each stage, several experiments are conducted for the recognition of colorectal carcinoma. The experimental results for all stages are presented in the following sections.

### 4.1. Stage 1: Experimentation of End-to-End CNNs

**Figure 9:**
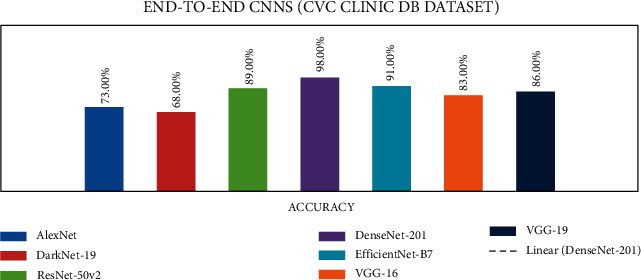
End-to-end CNN for CVC clinic DB graphical results.

**Figure 10:**
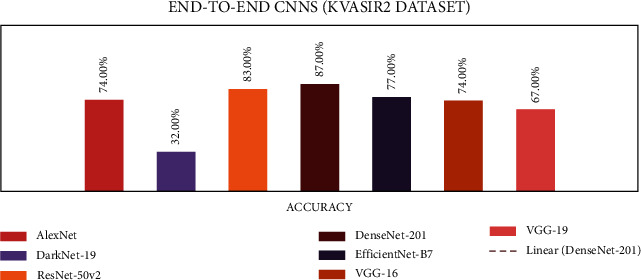
End-to-end CNN for Kvasir 2 graphical results.

**Figure 11:**
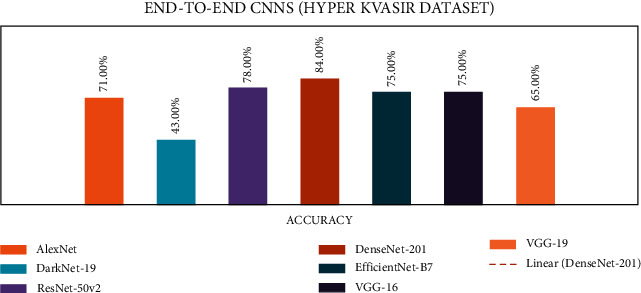
End-to-end CNN for Hyper Kvasir graphical results.

In stage 1, all the experiments are conducted for the seven end-to-end CNN models—AlexNet, DarkNet-19, ResNet50V2, DenseNet-201, EfficientNetB7, VGG-16, and VGG-19. All CNN models were trained with the CVC Clinic DB, Kvasir2, and Hyper Kvasir datasets. The experimental results are presented in Tables 7–9. The illustration of the results for all CNN models is presented using graphs and is shown in Figures 9–11.

According to the CVC clinic DB dataset results among all the seven CNN models, the DenseNet-201 achieved the highest accuracy of 98%.

According to the Kvasir 2 dataset results, the DenseNet-201 outperformed the six other CNN models with an accuracy of 87%.

According to the Hyper Kvasir dataset results, the DenseNet-201 outperformed the six other CNN models with an accuracy of 84%. Based on all datasets tested in stage 1, the DenseNet-201 CNN model showed the highest accuracy among all seven CNNs.

### 4.2. Stage 2: Experimentation of End-to-End CNNs and Fusion of CNNs with SVM

**Figure 12:**
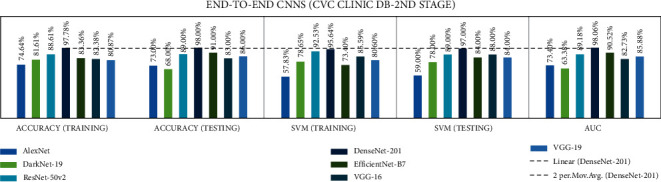
Comparing accuracies of end-to-end CNNs with CVC clinic DB dataset.

**Figure 13:**
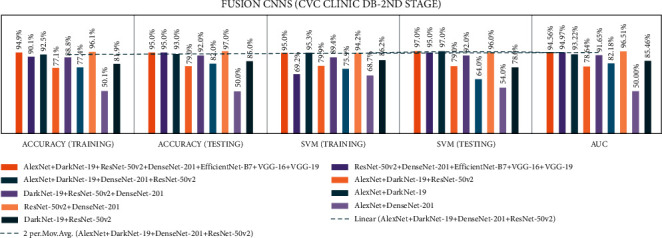
Comparing accuracies of fusion CNNs with CVC clinic DB dataset.

**Figure 14:**
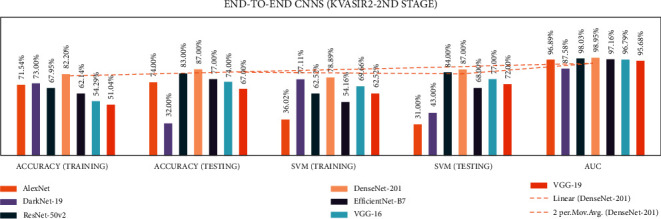
Comparing accuracies of end-to-end CNNs with Kvasir 2 dataset.

**Figure 15:**
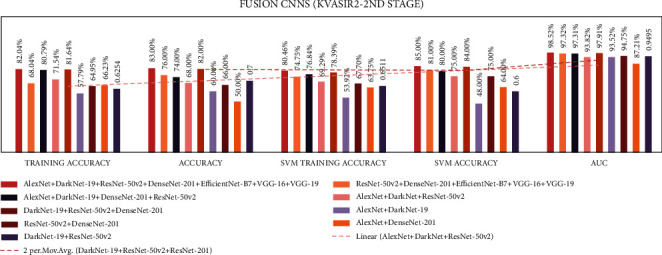
Comparing accuracies of fusion CNNs with Kvasir 2 dataset.

**Figure 16:**
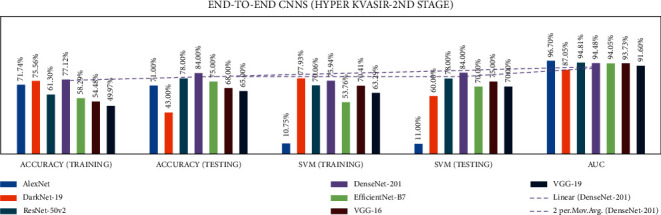
Comparing accuracies of end-to-end CNNs with Kvasir 2 dataset.

**Figure 17:**
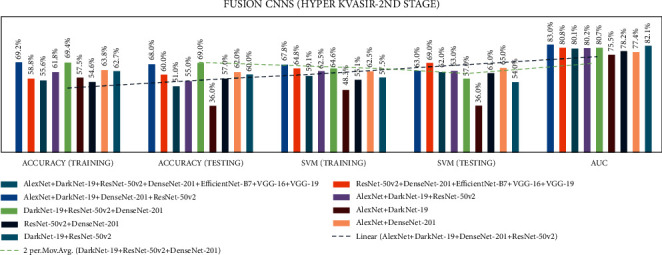
Comparing accuracies of fusion CNNs with Kvasir 2 dataset.

In stage two, experiments were performed with the seven end-to-end CNNs and nine fusion CNNs for all three datasets. Here, additionally, seven end-to-end CNNs and nine fusion CNNs were combined with the linear SVM classification. All experiment results are presented in Tables 10–15. The illustration of the second stage experimentation results is also presented in the graphs as shown in Figures 12–17.

According to the CVC Clinic DB dataset results among all the seven CNN models, the DenseNet-201 achieved the highest training, testing, SVM training, and SVM testing, and area under curve (AUC) results were 97.7%, 98.0%, 95.64%, 97.0%, and 98.06%, respectively.

According to the CVC Clinic DB dataset results, among all the nine fusion CNN models, the ADaDR-22 CNN fusion model achieved the highest training, testing, SVM training, and SVM testing, and the AUC results were 92.5%, 93.0%, 95.3%, 97.0%, and 93.22%, respectively.

In DenseNet-201, the nonpolyps class demonstrated lower performance with the support of 257 images, while the polyps class demonstrated 96% accuracy with 259 images. In the ADaDR-22 CNN fusion model, the nonpolyps class demonstrated lower performance with the support of 257 images, while the polyps class demonstrated 100% accuracy with 259 images.

According to the Kvasir 2 dataset results, among all the seven CNN models, the DenseNet-201 model achieved the highest training, testing, SVM training, and SVM testing, and AUC results with 82.2%, 87.0%, 78.89%, 87.0%, and 98.95%, respectively.

According to the Kvasir 2 dataset results among all the seven CNN models, the DaRD-22 CNN fusion model achieved the highest training, testing, SVM training, and SVM testing, and Area under the curve (AUC) with 81.6%, 82.0%, 78.39%, 84.0%, and 97.91%, respectively.

In DenseNet-201, some classes demonstrate lower performance accuracy with 300 images and normal-cecum polyps, and ulcerative-colitis class demonstrates equal and >90% accuracy with 300 images. In the DaRD-22 CNN fusion model, some classes demonstrate lower performance accuracy with 300 images, and normal-cecum, normal-pylorus, ulcerative-colitis classes demonstrate equal and >90% accuracy with 300 images.

In the Hyper Kvasir dataset, the DenseNet-201 model has the best training, testing, SVM training, and SVM Testing, and AUC results of 77.1, 84.0, 75.9, 84.0, and 94.48%, respectively.

In the Hyper Kvasir dataset, the DaRD-22 CNN fusion model achieved the highest training, testing, SVM training, and SVM testing, and AUC results of 69.4, 69.0, 64.6, 57.0, and 80.7%, respectively.

In the Hyper Kvasir dataset, the CNN DenseNet-201 model demonstrated no (zero) performance for some classes with fewer images. The classes bbps-0-1, bbps-2-3, cecum, pylorus, retroflex-stomach, and ulcerative-colitis-grade-3 demonstrated equal and >90% accuracy with 194,345,303,300,230 and 40 images, respectively. In Hyper Kvasir dataset, the DaRD-22 CNN fusion model shows no (zero) performance for some classes with fewer images. The classes polyps, pylorus, and retroflex-stomach demonstrated equal and >90% accuracy with 309,300, and 230 images, respectively.

### 4.3. Stage 3: Experimentation of End-to-End CNN + DWT + SVM

**Figure 18:**
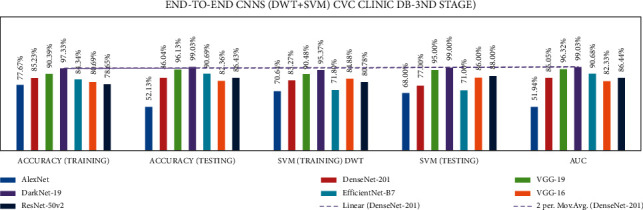
Comparing accuracies of DWT end-to-end with CVC clinic DB dataset.

**Figure 19:**
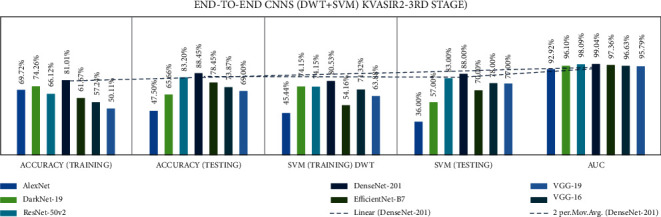
Comparing accuracies of DWT end-to-end with Kvasir 2 dataset.

**Figure 20:**
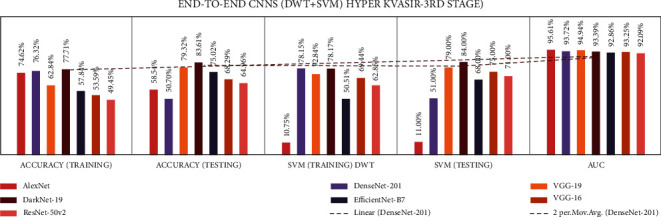
Comparing accuracies of DWT end-to-end with Hyper Kvasir dataset.

Stage three experiments involved seven end-to-end CNNs and nine fusion CNNs for all three datasets. Here transfer learning was applied to all seven end-to-end CNNs with DWT combined with the linear SVM classification. All experiment results are presented in Tables 16–18. The illustration of the second stage experimentation results is also presented in the graphs as shown in Figures 18–20.

According to the CVC Clinic dataset, the DenseNet-201 model achieved the highest accuracy for DWT-training, DWT-testing, DWT-SVM training, and DWT-SVM testing, and the DWT-Area under curve (AUC) results were 97.33, 99.03, 95.37, 99.0, and 99.03%, respectively.

According to the Kvasir 2 dataset, the DenseNet-201 model achieved the highest accuracy for DWT-training, DWT-testing, DWT-SVM training, and DWT-SVM testing, and the DWT-Area under curve (AUC) results were 81.01, 88.45, 80.53, 88.00, and 99.04%, respectively.

According to the Hyper Kvasir dataset, the DenseNet-201 model achieved the highest accuracy for DWT-training, DWT-testing, DWT-SVM training, and DWT-SVM testing, and the DWT-Area under curve (AUC) results were 77.71, 83.61, 78.17, 84.00, and 93.39%, respectively.

### 4.4. Stage 4: Experimentation of Fusion CNNs + DWT + SVM

**Figure 21:**
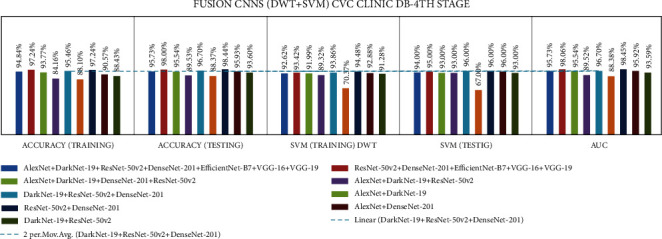
Comparing accuracies of DWT fusion CNN with CVC clinic DB dataset.

**Figure 22:**
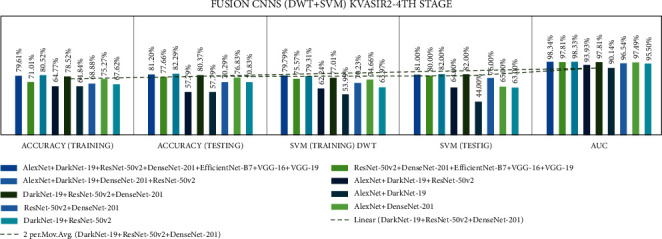
Comparing accuracies of DWT fusion CNN with Kvasir 2 dataset.

**Figure 23:**
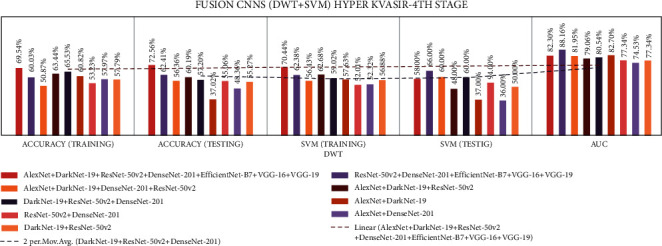
Comparing accuracies of DWT fusion CNN with Hyper Kvasir dataset.

Stage four experiment involved nine fusion CNNs for all three datasets. Here transfer learning was applied to all seven fusion CNNs with DWT using a combination of the linear SVM classification. The results of all the experimentations are presented in Tables 19–21. The illustration of the second stage experimentation results is also presented in graphs, as shown in Figures 21–23.

According to the CVC Clinic DB dataset, the DaRD-22 model achieved the highest DWT-training, DWT-testing, DWT-SVM training, and DWT-SVM testing, and the DWT-Area under curve (AUC) results were 95.46, 96.70, 93.86, 96.00, and 96.70%, respectively.

According to the Kvasir dataset, the DaRD-22 model achieved the highest DWT-training, DWT-testing, DWT-SVM training, and DWT-SVM testing, and the DWT-Area under curve (AUC) results were 78.52, 80.37, 77.01, 82.00, and 97.81%, respectively.

According to the Hyper Kvasir dataset, the ADaRDEV^2^-22 model achieved the highest DWT-training, DWT-testing, DWT-SVM training, and DWT-SVM testing, and the DWT-Area under curve (AUC) results were 69.54, 72.56, 70.44, 58.00, and 82.30%, respectively.

The results of the entire ColoRectalCADx system were compared with the three-stage GastroCADx proposed in 2021. Results for all three datasets are shown in Table 22. In GastroCADx, the system was compared with the four models from end-to-end CNN; however, in ColoRectalCADx, it was compared with seven models from end-to-end CNN. GastroCADx demonstrated that the ResNet-50 was the most suitable model, and for the ColoRectalCADx system, DenseNet-201 was the best model. By comparison, the two systems were almost identical, but the two differed in task behavior. Different system models such as Ensemble Classifier, DP-CNN, and MP-FSSD are discussed starting in 2021 and 2022 and compared with ColoRectalCADx. This proposed system obtained precisions of 98%, 88%, and 84%, respectively.

**Figure 24:**
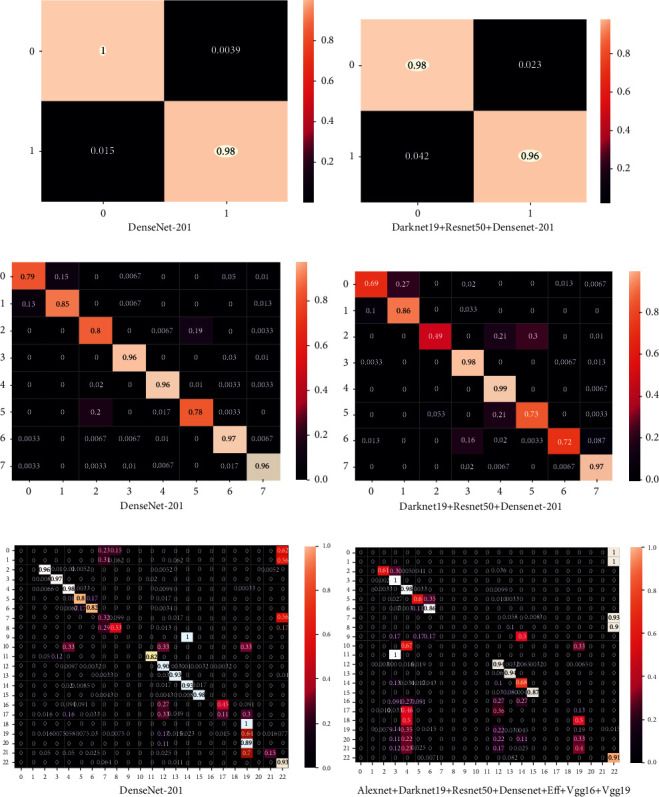
Confusion matrices of (a) CVC clinic DB dataset. (b) Kvasir 2. (c) Hyper Kvasir.

According to the classification results of the CVC Clinic DB, Kvasir2, and Hyper Kvasir datasets, the best accuracies were obtained with the DenseNet-201 for end-to-end CNNs. The CNN DaRD-22 and ADaRDEV^2^-22 fusion models were the most appropriate models for this proposed colorectal cancer identification system. The information accordingly provided with TP (True Positive), TN (True Negative), FP (False Positive), and FN (False Negative). The corresponding confusion matrices were formed based on the classes described for each dataset. CVC Clinic DB constituted 2 classes, Kvasir2 comprised 8 classes, and Hyper Kvasir comprised 23 classes. An *n* × *n* matrix summarizes the success of the predictions of a classification model, i.e., the correlation between the label and the classification of the model. A matrix formed as a confusion matrix indicates that every row is a real/true class and every column is a predicted/estimated class. The actual values were compared against the planned values. Therefore, for the right-hand side models, many elements are expected along the diagonal. Here, the confusion matrix was normalized, so the value of 1 was accepted as the highest value along the diagonal. Our model depicts that all the classes possess values near 1 along the diagonal. Using the high-performance CNNs with the particular dataset, it was observed that the confusion matrices with values near 1 have the best-classified classes. The high-performance CNN confusion matrices corresponding to the greatest accuracies for the classes are presented in Figure 24.

To estimate algorithm recognition performance, the algorithms with other medical motion colonoscopies image datasets were compared with CNN algorithms. The medical motion image recognition ratio results and the ROC curves of the different CNN algorithms obtained the best accuracies with the DenseNet-201 for end-to-end CNNs and fusion CNN's DarD-22 for the first two datasets and the Hyper Kvasir dataset ADaRDEV^2^-22 provided the highest accuracy. The ROC curves are presented and illustrated in Figure 14. These recognition rate curves, within this multi-class classification of the system, can be obtained at different accuracy levels. Based on the accuracies of the CNN and the ROC of the image classes, the accuracy class is represented and the class with the best accuracy is determined and presented in the graphs. These graphs are drawn against the TP (True Positive) rate and the FP (False Positive) rate.

Here, the CVC Clinic DB dataset, DenseNet-201 and the DaRD-22, presented approximately 99% to 100% accuracy of the two classes. Furthermore, in the Kvasir dataset, DenseNet-201 presented 99% to 100% accuracy and the DarD-22 approximately 97% to 100% accuracy given for eight classes. The Hyper Kvasir labeled dataset as DenseNet-201 presented 55% to 100% accuracy, and ADaRDEV^2^-22 presented approximately 23% to 100% accuracy given for 23 classes. In this integrated CNN, the four classes, which were misclassified, presented extremely inferior outputs.

**Figure 25:**
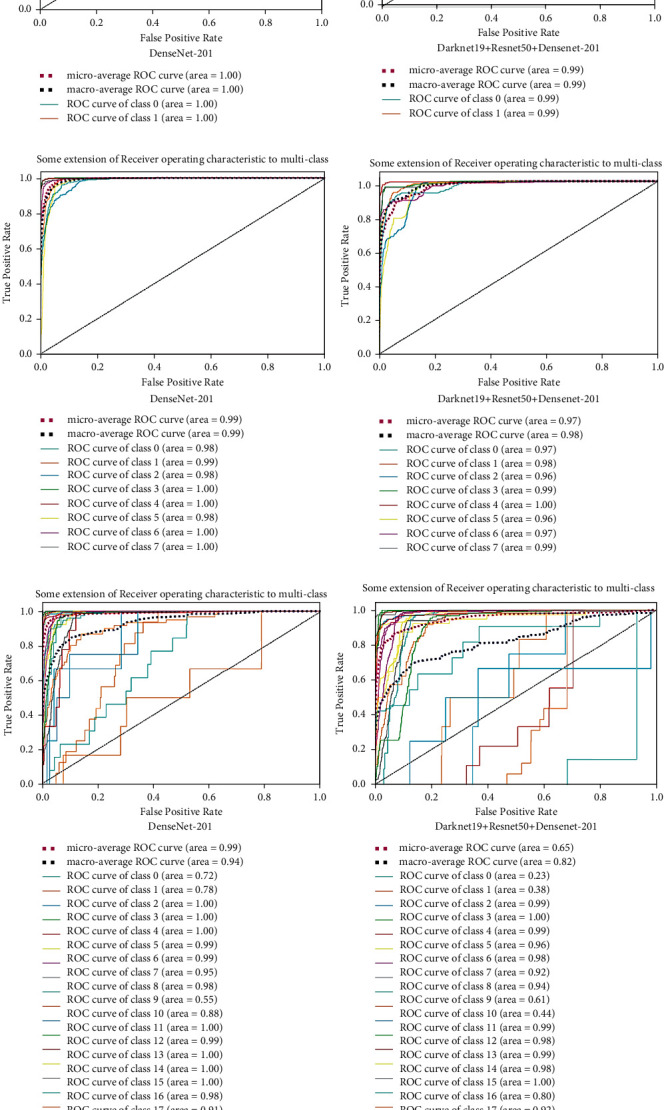
ROC curves of (a) CVC clinic DB dataset. (b) Kvasir 2. (c) Hyper Kvasir labeled.

The corresponding ROC curves for the three datasets are illustrated in Figure 25.

### 4.5. Stage 5: Semantic Segmentation Using UNet

This is the final stage of the ColoRectalCADx system for identifying and recognizing the real polyps, which are malignant, with the three types of the datasets: CVC Clinc-Seg, KvasirSeg, and Hyper Kvasir segmentation. The three datasets provide inputs to the ColoRectalCADx system, one after another, which is incorporated with the UNet CNN structure. The UNet works as the CNN with an encode-decoder network. A learning rate of 0.001 is provided, the batch size of the images is 64, and the number of epochs is 40. The resultant training and testing losses are presented in Table 23.

**Figure 26:**
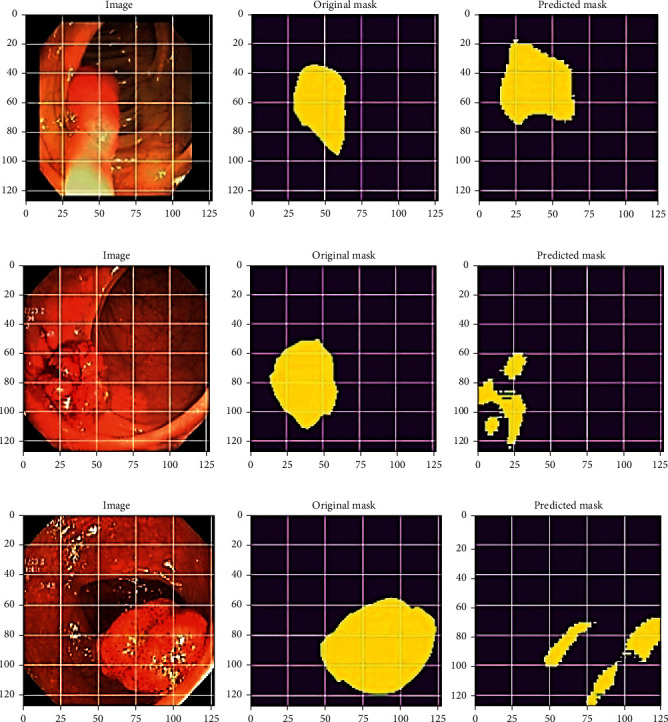
Semantic segmentation for predicted polyps. (a) CVC clinic DB. (b) Kvasir2. (c) Hyper Kvasir.

For each of the three datasets, the original images with the corresponding image masks of the malignant polyps are recognized accurately with training losses. The final predicted polyp obtained from the ColoRectalCADx system is shown in Figure 26.

**Figure 27:**
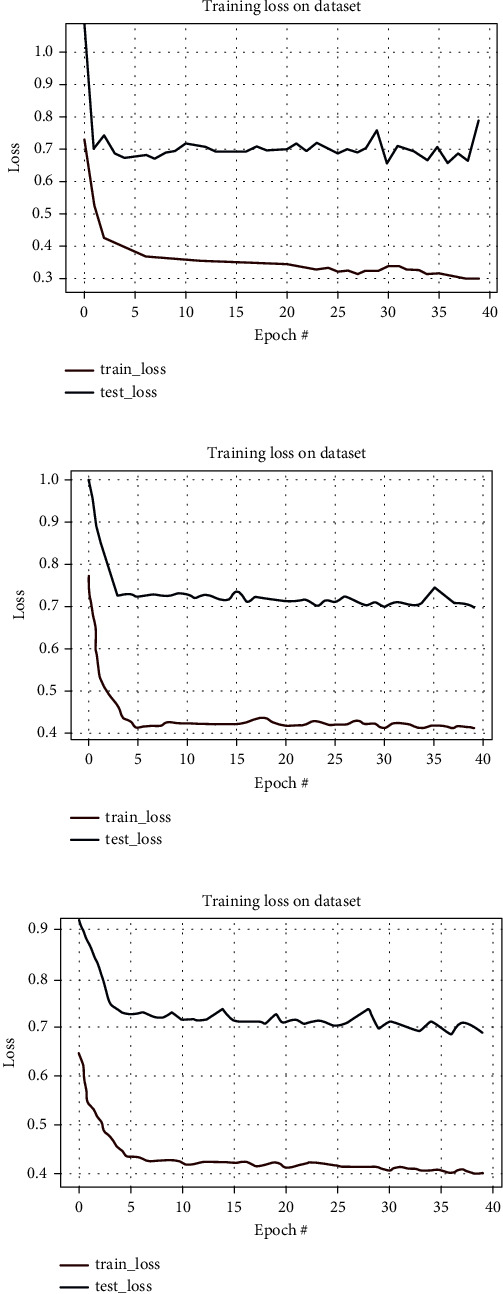
Loss graph. (a) CVC clinic DB. (b) Kvasir2. (c) Hyper Kvasir.

The system accurately and efficiently identified malignant polyps among all the input datasets with different polyps. The predicted polyp is the actual recognition of the malignant polyps. The corresponding loss and epochs graphs are shown in Figure 27.

## 5. Conclusion

This study explores how the three public datasets operate using the ColoRectalCaDx deep learning concept. The CVC Clinic DB, Kvasir, and Hyper Kvasir datasets are considered as inputs, and the system operates at five stages to obtain the results. The system starts at stage one with seven end-to-end CNNs such as AlexNet, DarkNet-19, ResNet50V2, DenseNet-201, EfficientNetB7, VGG-16, and VGG-19. Before proceeding to step two, the end-to-end CNNs are fused into nine different CNNs. In step two, end-to-end CNNs and fusion CNNs are transfer learned with SVM. In the third step of the system, the DWT is transfer learned with end-to-end CNNs to extract the spatial and temporal features from the CNN. The same features are also derived from the nine fusion CNNs in the fourth step. In this system, performance is achieved in stages as results are aggregated. The results presented in a tabular form are compared, and the best final CNN model is developed to identify colorectal carcinomas of the system. Experimentally, the results were obtained for the 5 stages. For each of the three datasets, from stage 1 to stage 3 end-to-end CNN, DenseNet-201 obtained the best testing accuracy (98%, 87%, 84%), ((98%, 97%), (87%, 87%), (84%, 84%)), ((99.03%, 99%), (88.45%, 88%), (83.61%, 84%)). For each of the three datasets, in stage 2, CNN DaRD-22 fusion obtained the best test accuracy ((93%, 97%) (82%, 84%), (69%, 57%)). And for stage 4, ADaRDEV^2^-22 fusion achieved the best test accuracy ((95.73%, 94%), (81.20%, 81%), (72.56%, 58%)). Once the results were achieved, the DenseNet-201 turned out to be the best end-to-end CNN model. The CNN DaRD-22 and ADaRDEV^2^-22 fusion models are the most appropriate models for this proposed colorectal cancer identification system. The final step of the system involves identifying malignant polyps in medical colonoscopy datasets. Among all three dataset images, semantic segmentation using the UNet CNN structure detects malignant polyps. The loss score for CVC clinic DB was 0.7842, for Kvasir2 by 0.6977, and Hyper Kvasir by 0.6910. Semantic segmentation identified polyps from the original frame with the intended malignant polyps.

In future work, we will consider applying the proposed system to all clinical colonoscopy motion video datasets. In the proposed system, the videos have multiple frames, thus such a video is represented with the highest number of images. These videos are represented in frame form. These images are categorized by perfect CNN and then visualize the polyps in colonoscopy motion videos with improved system representation for segmentation with good accuracy.

## Figures and Tables

**Table 1:** Literature survey.

Author	Year	Advantages	Limitations
Souaidi and Ansari [18]	2022	(i) To detect abnormalities in the polyp region of WCE and colonoscopy localization and visualization proposal.	(i) There is no discussion of the numerous CNN models.
		(ii) Here a detector for deep polyps, such as MP-FSSD, is suggested.	(ii) Only CNN models like VGG-16 are used.
		(iii) In this polyp detection work, VGG-16 backbones are used.	(iii) Only WCE and CVC clinic DB dataset are reviewed for polyp recognition.
Nisha and Palanisamy [19]	2022	(i) We automatically detect colorectal polyps with image enhancement.	(i) This method is only effective for a limited number of CNN models.
		(ii) The proposed work is the dual CNN path for classifying polyps and nonpolyps' patches in colonoscopy images.	(ii) This method is not discussed except with a CNN model.
		(iii) To enhance the image, the dual-path CNN and sigmoid classifier is used to efficiently detect polyps.	(iii) Only two sets of colonoscopy image data were proposed, such as CVC clinic DB and ETIS-Larib datasets.
		(iv) The proposed method is promising, and detects with accuracy of 99.60% and 90.81% with CVC clinic DB and ETIS-Larib datasets, respectively.	(iv) Images in the datasets are enhanced owing to which the accuracy of polyp detection will reduce.
			(v) The number of images is increased or live image datasets are used, and the suggested method for its operation is not addressed.
Guo et al. [20]	2022	(i) The two major challenges for the segmentation of colonoscopy image polyps are blurred boundaries and a close resemblance between the polyps and surrounding tissue.	(i) Here, five datasets are tested with a new UnX methodology, so it takes a long time to obtain the results.
		(ii) This system proposed a new transformer-based encounter network known as the uncertainty eXploration (UnX).	(ii) The precision levels of the results are good, but obtaining results is time-consuming.
		(iii) With this method, the system identified the uncertainty areas of polyps.	(iii) The complexity of the system is increased while comprehension of the system is much more tedious to a layman.
		(iv) This removes the uncertain elements of the images and emphatically recognizes the level of precision of malignant polyps.	(iv) There are inconsistent color distributions in the colonoscopy image system that displays poor results.
Yeung et al. [21]	2021	(i) The concept here is the segmentation of polyps and the identification of malignant polyps.	(i) With the five datasets, each image segmentation entails considerable time to obtain the results.
		(ii) The proposed method is CNN based on double attention for segmenting polyps using Focus-UNet.	(ii) Visualization quality may be good for certain datasets.
		(iii) This system combines efficient attention based on the spatial channel into a single focus gate selective deep learning of polyp characteristics.	(iii) The proposed focus-UNet system should have been upgraded to a lightweight design.
		(iv) Here for experimentation with the proposed methodology, inputs are provided using five colonoscopy datasets.	(iv) It is a complicated system.
		(v) The obtained results, such as the dice similarity coefficient, are 0.941 and 0910.	
Attallah and Sharkas [22]	2021	(i) Proposed a system called Gastro-CADx to classify several gastrointestinal diseases using deep learning approaches.	(i) Two datasets named dataset I and II, which are Kvasir and Hyper Kvasir, are used to assess the performance of Gastro-CADx.
		(ii) There are three phases to this system. These four different CNNs are used as feature extractors to extract spatial functionality.	(ii) However, this system has not been used on the numerous datasets.
		(iii) The properties extracted in the first stage are applied to the discrete wave transform (DWT) and the discrete cosine transform (DCT), which are used to extract temporal-frequency and spatial-frequency features.	(iii) The system is not even under discussion for the semantic segmentation concept for locating and identifying malignant polyps.
Jha et al. [23]	2021	(i) The design is the detection, localization, and segmentation of polyps in real-time.	(i) The system uses more than just a single dataset for experimentation and recognizing malignant polyps.
		(ii) This work calls for deep learning in technology.	(ii) The system provides moderate results (not highly accurate).
		(iii) The proposed solution to retrieve polyps from colonoscopy images developed ColonSegNet, which is a decoder-encoder architecture.	(iii) Architecture is complex to comprehend for laymen.
		(iv) detection, location, and segmentation are evaluated using standard computer vision measures.	
		(v) The system has a high processing rate of 182.38 frames per second.	
Ahraf et al. [24]	2020	(i) Suggested automated classification as a new technique for illustrating medical images using deep learning technology.	(i) Vast data of colonoscopy images are classified with different convolutional neural networks and the results are achieved differently.
		(ii) It helps to categorize the diverse medical images of several organs of the body.	(ii) The notions of interest are not addressed here and this has to be comprehensively addressed.
		(iii) It contains a summary of data and other health image classification applications, which support radiologists' efforts to improve diagnosis.	
Poudel et al. [25]	2020	(i) Provides a good architecture for classifying endoscopic images using an expanded efficient convolutional neural network.	(i) However, colorectal disorders are classified using convolutional neural networks.
		(ii) Proposed an architecture to classify endoscopic images using an effective convolutional neural network (CNN).	(ii) However, algorithms integrated with the various algorithms are compared with certain parameters.
		(iii) This is a highly accessible domain of assessing deeper layers by accumulating and reducing the expansion factor of spatial elements.	(iii) The results obtained are regarded as the most accurate and best algorithm for the identification of colorectal cancer (CRC).
		(iv) The investigator compares and evaluates the methodology using a variety of parameters.	
Zhou and Gao [26]	2019	(i) Here we discuss how CNN technologies enable intelligent recognition of medical motion images.	(i) However, there are no discussions on obtaining colorectal medical images from the colonoscopy screening images.
		(ii) Now large-scale intelligent recognition of medical motion images is assisted by CNN algorithms.	(ii) There is no explanation of the procedure to retrieve and categorize and then convert to results based on their image characteristics.
		(iii) Here, the features of the dense trajectory are initially learned followed by the features of depth, and the dense path functions are merged into the DL methods.	(iii) The techniques involved are time-consuming and require extensive computer statistics.
		(iv) Finally, extreme learning is functional in CNN where the descriptions of the bottom layer to the top layer are determined for medical image recognition	
Yang et al. [27]	2019	(i) Proposed a health-based device for categorizing and segmenting CT images for lung disease and hemorrhagic stroke, termed HTSCS for Health Images.	(i) This technique provides an advanced method of categorization and segmentation using art.
		(ii) Internet Health of-Things (IHoT) uses transferable model learning, based on deep learning concepts with traditional methodologies for the best precision for medical image classification and segmentation	(ii) This Internet of medical Things has worked with various IoT devices with the connection of computed tomography devices.

**Table 2:** Fusion models and their suggested names.

Fusion model	Suggested name
AlexNet + DarkNet-19 + ResNet-50v2 + DenseNet-201 + Efficientnet-B7 + VGG-16 + VGG-19	ADaRDEV^2^-22
ResNet-50v2 + DensNet-201 + EfficientNet-B7 + VGG-16 + VGG19	RDEV^2^-22
AlexNet + DarkNet-19 + DenseNet-201 + ResNet-50V2	ADaDR-22
AlexNet + DarkNet-19 + ResNet-50V2	ADaR-22
DarkNet-19 + ResNet-50V2 + DenseNet-201	DaRD-22
AlexNet + DarkNet-19	ADa-22
ResNet-50V2 + DenseNet-20	RD-22
AlexNet + DenseNet-201	AD-22
DarkNet-19 + ResNet-50V2	DaR-22

**Table 3:** The number of parameters of CNNs.

CNN architecture models		Introduced year	Total params	Trainable params	Nontrainable params	Layers
AlexNet [47, 48]		2012	2,81,02,775	2,80,81,639	21,136	23
DarkNet-19 [49]		2017	1,60,45,847	1,60,32,983	12,864	19
ResNet-50v2 [50, 51]		2016	2,59,33,975	23,69,175	2,35,64,800	50
DenseNet-201 [52, 53]		2018	1,94,29,463	11,07,479	1,83,21,984	201
Efficientnet-B7 [54]		2019	6,55,73,799	14,76,119	6,40,97,680	813
VGG-16 [55]		2014	1,53,14,391	5,99,703	1,47,14,688	16
VGG-19 [56]		2014	2,06,24,087	5,99,703	2,00,24,384	13
Proposed fusion models	ADaRDEV^2^-22	2022	19,10,28,063	7,02,94,911	12,07,33,152	
	RDEV^2^-22		14,68,78,383	2,61,79,231	12,06,99,152	
	ADaDR-22		8,94,87,664	4,75,66,880	4,19,20,784	
	ADaR-22		2,59,33,000	23,68,200	2,35,64,800	
	DaRD-22		6,13,99,840	1,95,00,192	4,18,99,648	
	ADa-22		4,41,26,048	4,40,92,048	34,000	
	RD-22		4,53,61,624	34,74,840	4,18,86,784	
	AD-22		4,75,16,384	2,91,73,264	1,83,43,120	
	DaR-22		4,19,67,694	1,83,90,030	2,35,77,664	

**Table 4:** System specifications.

System	Precision tower T5810
Company	Dell
Processor	Intel® Xeon® CPU core i7 E5-2630
Speed	2.20 GHz
RAM	32 GB
GPU	GPU NVIDIA Xp.
Software environment	Google Colab Pro with python 3.7.12
Software Python packages	Keras and TensorFlow 2.7.0

**Table 5:** Training and testing split of three datasets.

Datasets	Training set	Validation set	Test sets	Total images
CVC-clinic DB [32]	900	102	516	1518
Kvasir2 [33, 34].	5120	480	2400	8000
Hyper Kvasir Labeled [35, 36].	7470	634	2577	10681

**Table 6:** Hyperparameters for colorectalCADx system.

Dataset	Epochs	Batch sizes	Learning rate	Optimizer	Momentum	Dropout
CVC clinic DB	10	16	0.0001	sgd	0.9	0.5
Kvasir2	10	64	0.0001	sgd	0.9	0.5
Hyper Kvasir labeled	10	64	0.0001	sgd	0.9	0.5

**Table 7:** End-to-end CNN for CVC clinic DB.

End-to-end CNNs	Accuracy in %
AlexNet	73.00
DarkNet-19	68.00
ResNet-50v2	89.00
DenseNet-201	98.00
Efficientnet-B7	91.00
VGG-16	83.00
VGG-19	86.00

**Table 8:** End-to-end CNN for Kvasir 2.

End-to-end CNNs	Accuracy in %
AlexNet	74.00
DarkNet-19	32.00
ResNet-50v2	83.00
DenseNet-201	87.00
Efficientnet-B7	77.00
VGG-16	74.00
VGG-19	67.00

**Table 9:** End-to-end CNN for hyper kvasir.

End-to-end CNNs	Accuracy in %
AlexNet	71.00
DarkNet-19	43.00
ResNet-50v2	78.00
DenseNet-201	84.00
Efficientnet-B7	75.00
VGG-16	75.00
VGG-19	65.00

**Table 10:** Comparison accuracies of the end-to-end and fusion CNNs of CVC clinic DB dataset.

	Accuracy in % (training)	Accuracy in % (testing)	SVM in % (training)	SVM in % (testing)	AUC in %
*End-to-end CNNs*					
AlexNet	74.64	73.00	57.83	59.00	73.40
DarkNet-19	81.61	68.00	78.65	78.00	68.38
ResNet-50v2	88.61	89.00	92.53	89.00	89.18
DenseNet-201	97.78	98.00	95.64	97.00	98.06
Efficientnet-B7	83.36	91.00	73.40	84.00	90.52
VGG-16	82.38	83.00	85.59	88.00	82.73
VGG-19	80.87	86.00	80.60	84.00	85.88

*Fusion CNNs*					
ADaRDEV^2^-22	94.9	95.0	95.0	97.0	94.56
RDEV^2^-22	90.1	95.0	69.2	95.0	94.97
ADaDR-22	92.5	93.0	95.3	97.0	93.22
ADaR-22	77.1	79.0	79.9	79.0	78.64
DaRD-22	88.8	92.0	89.4	92.0	91.65
ADa-22	77.4	82.0	75.9	64.0	82.18
RD-22	96.1	97.0	94.2	96.0	96.51
AD-22	50.1	50.0	68.7	54.0	50.00
DaR-22	81.9	85.0	86.2	78.0	85.46

**Table 11:** Comparison of precision and support of CVC clinic DB classes.

Classes	High performed CNN models		
	DenseNet-201	ADaDR-22	Support
	Precision	Precision	
Nonpolyps	0.97	0.91	257
Polyps	1	0.96	259

**Table 12:** The comparison accuracies of the end-to-end and fusion CNNs of Kvasir 2 dataset.

	Accuracy in % (training)	Accuracy in % (testing)	SVM in % (training)	SVM in % (testing)	AUC in %
*End-to-end CNNs*					
AlexNet	71.54	74.00	36.02	31.00	96.89
DarkNet-19	73.00	32.00	77.11	43.00	87.58
ResNet-50v2	67.95	83.00	62.52	84.00	98.03
DenseNet-201	82.20	87.00	78.89	87.00	98.95
Efficientnet-B7	62.14	77.00	54.16	68.00	97.16
VGG-16	54.29	74.00	69.66	77.00	96.79
VGG-19	51.04	67.00	62.52	72.00	95.68

*Fusion CNNs*					
ADaRDEV^2^-22	82.04	83.00	80.46	85.00	98.52
RDEV^2^-22	68.04	76.00	74.75	81.00	97.32
ADaDR-22	80.79	74.00	76.84	80.00	97.31
ADaR-22	71.54	68.00	69.29	75.00	93.82
DaRD-22	81.64	82.00	78.39	84.00	97.91
ADa-22	57.79	60.00	53.92	48.00	93.52
RD-22	64.95	66.00	67.70	75.00	94.75
AD-22	66.23	50.00	63.75	64.00	87.21
DaR-22	62.54	70.00	65.11	60.00	94.95

**Table 13:** Comparison of precision and support of Kvasir 2 classes.

Classes	High performed CNN model		
	DenseNet-201	DaRD-22	Support
	Precision	Precision	
Dyed-lifted-polyps	0.78	0.81	300
Dyed-resection-margins	0.87	0.82	300
Esophagitis	0.79	0.73	300
Normal-cecum	0.97	0.92	300
Normal-pylorus	0.91	0.95	300
Normal-*z*-line	0.78	0.72	300
Polyps	0.9	0.76	300
Ulcerative-colitis	0.93	0.92	300

**Table 14:** Comparing accuracies of the end-to-end and fusion CNNs of Hyper Kvasir dataset.

	Accuracy in % (training)	Accuracy in % (testing)	SVM in % (training)	SVM in % (testing)	AUC in %
*End-to-end CNNs*					
AlexNet	71.74	71.00	10.75	11.00	96.70
DarkNet-19	75.56	43.00	77.93	60.00	87.05
ResNet-50v2	61.30	78.00	71.06	78.00	94.81
DenseNet-201	77.12	84.00	75.94	84.00	94.48
Efficientnet-B7	58.29	75.00	53.76	70.00	94.05
VGG-16	54.48	68.00	70.41	75.00	93.73
VGG-19	49.97	65.00	63.29	70.00	91.60

*Fusion CNNs*					
ADaRDEV^2^-22	69.2	68.0	67.8	63.0	83.0
RDEV^2^-22	56.8	60.0	64.8	69.0	80.8
ADaDR-22	55.6	51.0	59.1	62.0	80.1
ADaR-22	61.8	55.0	62.5	63.0	80.2
DaRD-22	69.4	69.0	64.6	57.0	80.7
ADa-22	57.5	36.0	48.3	36.0	75.5
RD-22	54.6	57.0	56.1	61.0	78.2
AD-22	63.8	62.0	62.5	65.0	77.4
DaR-22	62.7	60.0	57.5	54.0	82.1

**Table 15:** Comparison of precision and support of Hyper Kvasir classes.

Classes	High performed CNN models		
	DenseNet-201	DaRD-22	Support
	Precision	Precision	
Barretts	0	0	13
Barretts-short-segment	0	0	16
Bbps-0-1	0.93	0.87	194
Bbps-2-3	0.96	0.87	345
Cecum	0.9	0.51	303
Dyed-lifted-polyps	0.81	0.21	301
Dyed-resection-margins	0.81	0.48	297
Esophagitis-a	0.46	0	121
Esophagitis-b-d	0.63	0	78
Hemorrhoids	0	0	6
Ileum	0	0	3
Impacted-stool	0.85	0	40
Polyps	0.82	0.93	309
Pylorus	0.94	0.91	300
Retroflex-rectum	0.89	0.86	117
Retroflex-stomach	0.99	0.99	230
Ulcerative-colitis-grade-0-1	0	0	11
Ulcerative-colitis-grade-1	0.5	0	61
Ulcerative-colitis-grade-1-2	0	0	4
Ulcerative-colitis-grade-2	0.54	0.69	133
Ulcerative-colitis-grade-2-3	0	0	9
Ulcerative-colitis-grade-3	1	0	40
*z*-line	0.69	0.53	280

**Table 16:** Comparison accuracies of the end-to-end of CVC clinic DB dataset.

DWT					
End-to-end CNN	Accuracy in % (training)	Accuracy in % (testing)	SVM in % (training)	SVM in % (testing)	AUC in %
AlexNet	77.67	52.13	70.64	68.00	51.94
DarkNet-19	85.23	86.04	83.27	77.00	86.05
ResNet-50v2	90.39	96.13	90.48	95.00	96.32
DenseNet-201	97.33	99.03	95.37	99.00	99.03
Efficientnet-B7	84.34	90.69	71.80	71.00	90.68
VGG-16	80.69	82.36	84.88	86.00	82.33
VGG-19	78.65	86.43	80.78	88.00	86.44

**Table 17:** Comparison accuracies of the end-to-end Kvasir 2 dataset.

DWT					
End-to-end CNN	Accuracy in % (training)	Accuracy in % (testing)	SVM in % (training)	SVM in % (testing)	AUC in %
AlexNet	69.72	47.50	45.44	36.00	92.92
DarkNet-19	74.26	65.66	74.15	57.00	96.10
ResNet-50v2	66.12	83.20	74.15	83.00	98.09
DenseNet-201	81.01	88.45	80.53	88.00	99.04
Efficientnet-B7	61.67	78.45	54.16	70.00	97.36
VGG-16	57.24	73.87	71.32	78.00	96.63
VGG-19	50.11	69.00	63.83	77.00	95.79

**Table 18:** Comparison accuracies of the end-to-end of Hyper Kvasir dataset.

DWT					
End-to-end CNN	Accuracy in % (training)	Accuracy in % (testing)	SVM in % (training)	SVM in % (testing)	AUC in %
AlexNet	74.62	58.54	10.75	11.00	95.61
DarkNet-19	76.32	50.70	78.15	51.00	93.72
ResNet-50v2	62.84	79.32	72.84	79.00	94.94
DenseNet-201	77.71	83.61	78.17	84.00	93.39
Efficientnet-B7	57.84	75.02	50.51	68.00	92.86
VGG-16	53.59	68.29	69.44	75.00	93.25
VGG-19	49.45	64.96	62.85	71.00	92.09

**Table 19:** The comparison accuracies of the fusion CNNs of the CVC clinic DB dataset.

DWT					
Fusion CNNs	Accuracy in % (training)	Accuracy in % (testing)	SVM in % (training)	SVM in % (testing)	AUC in %
ADaRDEV^2^-22	94.84	95.73	92.62	94.00	95.73
RDEV^2^-22	97.24	98.00	93.42	95.00	98.06
ADaDR-22	93.77	95.54	91.99	93.00	95.54
ADaR-22	84.16	89.53	89.32	93.00	89.52
DaRD-22	95.46	96.70	93.86	96.00	96.70
ADa-22	83.10	88.37	70.37	67.00	88.38
RD-22	97.24	98.44	94.48	96.00	98.45
AD-22	90.57	95.93	92.88	96.00	95.92
DaR-22	88.43	93.60	91.28	93.00	93.59

**Table 20:** Comparison accuracies of the fusion CNNs of the Kvasir 2 dataset.

DWT					
Fusion CNNs	Accuracy in % (training)	Accuracy in % (testing)	SVM in % (training)	SVM in % (testing)	AUC in %
ADaRDEV^2^-22	79.61	81.20	79.79	81.00	98.34
RDEV^2^-22	71.01	77.66	75.57	80.00	97.81
ADaDR-22	80.52	82.29	79.31	82.00	98.33
ADaR-22	64.77	57.79	62.24	64.00	93.93
DaRD-22	78.52	80.37	77.01	82.00	97.81
ADa-22	64.84	57.79	53.99	44.00	90.14
RD-22	68.88	70.29	70.23	76.00	96.54
AD-22	75.27	76.83	74.66	65.00	97.49
DaR-22	67.62	70.83	63.97	63.00	95.50

**Table 21:** Comparison accuracies of the fusion CNNs of the Hyper Kvasir dataset.

DWT					
Fusion CNNs	Accuracy in % (training)	Accuracy in % (testing)	SVM in % (training)	SVM in % (testing)	AUC in %
ADaRDEV^2^-22	69.54	72.56	70.44	58.00	82.30
RDEV^2^-22	60.0%	62.41	62.38	66.00	88.16
ADaDR-22	50.87	56.36	56.43	60.00	81.95
ADaR-22	63.44	60.19	62.68	48.00	79.06
DaRD-22	65.53	57.20	59.02	60.00	80.54
ADa-22	60.82	37.02	57.63	37.00	82.70
RD-22	53.23	55.96	52.01	54.00	77.34
AD-22	57.97	48.36	52.32	36.00	74.53
DaR-22	57.79	55.27	56.88	50.00	77.34

**Table 22:** Comparison of results related studies for CVC clinic DB, Kvasir 2 , and Hyper Kvasir datasets with previous state-of-the-art methods.

Dataset	Author	Method	Accuracy in %
CVC clinic DB	Attallah and Sharkas [22]	GastroCADx	—
	Liew et al. [71]	Ensemble classifier (ResNet50+Adaboost)	97.91
	Sharma et al. [72]	Ensemble classifier	98.3
	Nisha and Palanisamy [19]	DP-CNN	99.60
	Souaidi and Ansari [18]	MP-FSSD	91.56
	Ours	ColoRectalCADx (proposed)	99.00

Kvasir2	Attallah and Sharkas [22]	GastroCADx	97.3
	Sharma et al. (2022) [72]	Ensemble classifier	97
	Ours	ColoRectalCADx (proposed)	88.00

Hyper Kvasir	Attallah and Sharkas [22]	GastroCADx	99.7
	Ours	ColoRectalCADx (proposed)	84.00

**Table 23:** Parameters of UNet for semantic segmentation.

Dataset	Epochs	Learning rate	Batch size	Train loss	Test loss	Total time taken for model (s)
CVC clinic DB	40	0.001	64	0.2998	0.7842	862.61
Kvasir2	40	0.001	64	0.412	0.6977	1477.32
Hyper Kvasir	40	0.001	64	0.4005	0.691	1374.04

## Data Availability

Data are publicly available from the following websites: Dataset 1 was obtained from https://www.kaggle.com/datasets/balraj98/cvcclinicdb. Dataset 2 was obtained fromhttps://datasets.simula.no/kvasir/. Dataset 3 was obtained from https://datasets.simula.no/hyper-kvasir/.
